# Implementation of a Surgical Safety Checklist: Interventions to Optimize the Process and Hints to Increase Compliance

**DOI:** 10.1371/journal.pone.0116926

**Published:** 2015-02-06

**Authors:** Gerald Sendlhofer, Nina Mosbacher, Leitgeb Karina, Brigitte Kober, Lydia Jantscher, Andrea Berghold, Gudrun Pregartner, Gernot Brunner, Lars Peter Kamolz

**Affiliations:** 1 Executive Department for Quality and Risk Management, University Hospital Graz, Graz, Austria; 2 Division of Plastic, Aesthetic and Reconstructive Surgery, Department of Surgery, Medical University Graz, Graz, Austria; 3 Institute for Medical Informatics, Statistics and Documentation, Medical University Graz, Graz, Austria; 4 University Hospital Graz, Graz, Austria; McGill University, CANADA

## Abstract

**Background:**

A surgical safety checklist (SSC) was implemented and routinely evaluated within our hospital. The purpose of this study was to analyze compliance, knowledge of and satisfaction with the SSC to determine further improvements.

**Methods:**

The implementation of the SSC was observed in a pilot unit. After roll-out into each operating theater, compliance with the SSC was routinely measured. To assess subjective and objective knowledge, as well as satisfaction with the SSC implementation, an online survey (N = 891) was performed.

**Results:**

During two test runs in a piloting unit, 305 operations were observed, 175 in test run 1 and 130 in test run 2. The SSC was used in 77.1% of all operations in test run 1 and in 99.2% in test run 2. Within used SSCs, completion rates were 36.3% in test run 1 and 1.6% in test run 2. After roll-out, three unannounced audits took place and showed that the SSC was used in 95.3%, 91.9% and 89.9%. Within used SSCs, completion rates decreased from 81.7% to 60.6% and 53.2%. In 2014, 164 (18.4%) operating team members responded to the online survey, 160 of which were included in the analysis. 146 (91.3%) consultants and nursing staff reported to use the SSC regularly in daily routine.

**Conclusion:**

These data show that the implementation of new tools such as the adapted WHO SSC needs constant supervision and instruction until it becomes self-evident and accepted. Further efforts, consisting mainly of hands-on leadership and training are necessary.

## Introduction

The importance of a strong safety culture that enhances patient safety initiatives has been reiterated for years in the healthcare system [1–3]. In 2009, the University Hospital Graz, a 1,500-bed tertiary university hospital and the second largest in Austria with more than 1,200,000 outpatients and 88,000 inpatients per year, began implementing comprehensive patient safety tools through the systematic introduction of clinical risk management into routine hospital procedures [4].

One of the key elements in a risk management approach to safer hospital environments is the use of a surgical safety checklist (SSC) [5, 6]. The SSC is an inexpensive tool capable of shifting the hierarchical culture in the operating room and fostering patient safety attitudes [7–9]. Approximately 234 million operations are performed annually and with regard to surgical procedures the WHO SSC has shown the potential to be effective at reducing complication and mortality rates. Furthermore, positive effects on communication procedures can also be expected from using a structured tool such as the SSC [7,10–15]. By now, more than 4,000 hospitals worldwide have implemented the SSC [16] and, on the advice of the WHO, modified it according to local circumstances. For example, the WHO SSC has 19 items and is checked at three distinct checkpoints, however, the effect of adaption with respect to compliance remains unclear [15, 16–18].

The functioning of a checklist requires people to make one salient change in their routine procedures; in particular, the operating theater team has to pause [6] during the team time out (TTO) and sign out (SO) phases before continuing. Though positive reports are available on the use of the SSC, it is still not self-evident that the SSC will be accepted and used as intended by the WHO [16]. Multiple barriers such as misuse, nonuse or incomplete execution are reported and reduce chances for the best possible outcomes [15]. Reasons for this can lie in a lack of positive role models or less than enthusiastic team members, hierarchical barriers, limited knowledge of correct usage and inappropriate implementation procedures [16]. Thereby, active involvement in the implementation phase as well as continuous evaluation and training is presumed to greatly impact the compliance and acceptance by all team members [15, 19, 20].

Our hospital performs approximately 47,000 surgical procedures each year. In 2011, we began to implement an adapted WHO SSC in a piloting unit and introduced the SSC thereafter into all 44 operating theaters over an implementation period of one year. Compliance with checklist use is a necessary prerequisite for working effectively [21], therefore, next to outcome parameters such as adverse events, observing the use of the SSC is essential. Compliance to the SSC provides clues about the acceptance of the SSC in a team.

The primary aim of this study was to evaluate SSC compliance within a tertiary university hospital using a hospital-wide and adapted SSC within 44 operating theaters. The secondary aim was to assess healthcare professionals’ knowledge of and satisfaction with the SSC to receive further hints to improve SSC compliance. To the best of our knowledge this is the first report from Austria concerning compliance, satisfaction and employee knowledge with respect to the SSC in a tertiary hospital.

## Methods

### Adapting the SSC

In 2011, an interdisciplinary team consisting of anesthesiologists, surgeons, nurse anesthetists, operating theater nurses, legal representatives and quality managers adapted the WHO SSC to local circumstances. For example, we separated the item “patient identity” from the original WHO SSC during the SI and TTO into two distinct items in each SSC phase, namely “patient name” and “patient birth date”. We assumed that each aspect that helps to correctly identify the patient requires time and proper alertness and should not be combined into one general item. Furthermore, the backside of the SSC contained directives to help guide the operating staff through the correct SSC procedure.

The adapted SSC consisted of three phases. The sign-in (SI) phase, before induction of anesthesia, had to be checked by the operating theater nurse, nurse anesthetists and the anesthesiologist. The TTO as well as the SO had to be initiated by the surgeon. The circulating nurse as the designated checklist coordinator then guided the team throughout all questions and ticked the checkboxes. The checklist coordinator was obliged to only tick the checkbox if an answer was given to the corresponding question. Finally, the SSC becomes part of the patient’s paper-based notes.

Prior to the first attempt to use the SSC in the pilot unit (test run 1), the corresponding operating teams were given an introduction through a YouTube video from the NHS entitled “How not to do the surgical safety checklist” [22]. They were also provided training on how to ask and answer questions.

### Implementing the SSC

In the pilot unit, test run 1of the SSC was supervised by quality managers present in the operating theater for one week to ensure correct usage as well as questioning and answering procedures (June 2011). After one month, all SSCs were collected and SSC use for each operation was noted, as was whether checkboxes were ticked as required. Results were discussed with the operating team and department leaders. Any indications that items needed to be changed were implemented into the second version of the SSC. Thereafter, the SSC was used for another four months in the pilot unit and re-observed in November 2011 by collecting all available SSC copies over a one-month period (test run 2). Further improvements were then implemented into the SSC and in the end the SSC consisted of 14 SI items, 13 TTO items and 8 SO items.

Starting in December 2011, other operating staffs were prepared for SSC introduction in their areas and trained in its usage (pediatric surgery, trauma surgery, ophthalmic surgery, surgery (general, thoracic, vascular, plastic, transplant and cardiac surgery), dermatologic surgery, gynecology and obstetrics, head and neck surgery, neurosurgery, urology, oral and cranial maxillofacial surgery). Approximately 50% out of 900 employees working in operating theaters at that time were reached through training, role play or focus group discussions. We also identified contact persons in each area to support employees if questions or uncertainties arose during the implementation. By December 2012, all surgical departments used the SSC.

### Assessing compliance

**Fig 1:**
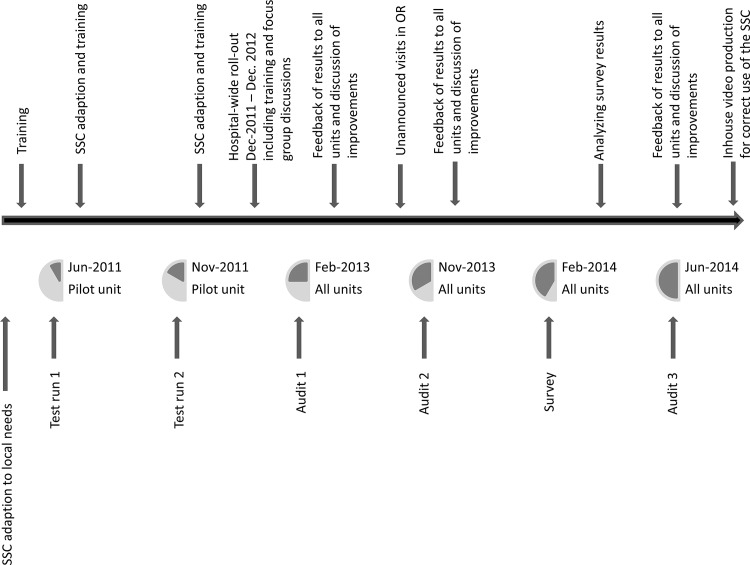
Details on the chronology of the implementation, modification and intervention process.

To assess the SSC compliance rate within each department, unannounced audits have been introduced. We tried to identify responders and non-responders of the SSC for further improvement cycles, which consisted mainly of further focus group discussions or trainings with the operating staff. Within the first audit in February 2013, users of the SSC were asked to recall the number of operations for two given days together with the numbers of completed, partially completed and missing SSCs. The days were determined and announced via email by the Department of Quality and Risk Management to all senior managers of surgical departments. For the second and third audit in November 2013 and June 2014 again two days were determined and announced via email, however, SSCs were collected and compared to performed operations by the Department of Quality and Risk Management. The numbers of performed operations versus the number of collected SSCs were matched with scheduled and definitely performed operations. Corresponding data were collected from hospital’s electronic documentation system. Fig. 1 shows details on the chronology of the implementation, modification and intervention process.

### Online Survey

In 2014, an online survey (Table 1) assessing use of, satisfaction with and knowledge of the SSC was conducted. We used a Swiss survey recently published by Mascherek et al. This survey was validated by the Patient Safety Foundation in Switzerland [20]. In the Swiss version of the questionnaire, 10 (4 true and 6 false) statements referring to the SSC were given; one question was adapted to local circumstances which finally resulted in 3 true and 7 false items. The online questionnaire was sent to all 891 employees working in one of the 44 operating theaters, a sample that corresponds to 20.1% of the total workforce of the university hospital and includes all professional groups. Email addresses were obtained from the in-house mailing list. Employees were informed about the aim of the survey and were invited to participate. Furthermore, it was pointed out that all collected data was going to be stored in the Department of Quality and Risk Management and that data analysis would be strictly anonymous. The online survey was open for one month and after two weeks a system reminder was sent to non-responders. Each participant was given a transaction authentication number (TAN) using the software Evasys, Healthcare Survey Automation Suite. Each TAN could only be used once and therefore each person was able to participate only once. Employees had the free choice to decline participation or to withdraw from the survey at any time. Participants were also given the possibility to skip questions if they felt uncomfortable answering them. The conduct of the online survey was approved by the Medical University Graz Ethics Committee (vote-number: 26-137 ex 13/14).

**Table 1:** Questionnaire items: general use of the SSC, frequency of SSC use, satisfaction with the implementation as well as subjective and objective knowledge (correct answer in brackets) as used in the Swiss survey [20].

	Do you use a Surgical Safety Checklist to support patient safety?
General use of an SSC	Which of the following checklists do you know?
	- WHO Surgical Safety Checklist
	- Universal Protocol of Joint Commission (JCAHO)
	- Recommendations of the Patient Safety Foundation Switzerland
	- None of these
Frequency of SSC use	In how many operations do you use the Surgical Safety Checklist at your primary working place?
	- Never or almost never (0–10% of operations)
	- Rarely (11–30% of operations)
	- Occasionally (31–50% of operations)
	- Frequently (51–70% of operations)
	- Most of the time (71–90% of operations)
	- Always or almost always (91–100% of operations)
Satisfaction with SSC	How satisfied are you with the implementation of the Surgical Safety Checklist at your primary working place?
	- Very satisfied
	- Satisfied
	- Somewhat satisfied
	- Rather unsatisfied
	- Very unsatisfied
Subjective knowledge	How do you rank your knowledge with respect to the content and utilization of the Surgical Safety Checklist?
	- Very good
	- Rather good
	- Okay
	- Rather bad
	- Very bad
Objective knowledge	Questions
	The SSC is a synonym for Team Time Out. (false)
	The SSC must not be completed by all team members. (false)
	The SSC requires exact documentation of the number of used sponges. (false)
	The SSC exclusively addresses surgeons. (false)
	The SSC recommends an antibiotic prophylaxis within 60 minutes of surgery. (true)
	The SSC shall support inexperienced members of the team. (false)
	The SSC is a tool used to attribute mistakes and misses to specific persons. (false)
	The SSC aims to prevent accidental omissions within routine procedures. (true)
	The SSC aims to improve team communication. (true)
	The SSC may be used to document complications. (false)

Question 2 of objective knowledge was changed to adhere to local procedures.

### Outcome Measures

The primary outcome measures were whether the SSC was generally used and the respective completion rate in case of using a SSC. Secondary outcome measures were knowledge regarding the SSC as well as satisfaction with its implementation and the frequency of use as self-reported by the respondent.

### Statistical analysis

The survey data was analyzed using descriptive statistics for the total cohort and for each of the two professional groups (consultants and nursing staff). Categorical variables are presented as absolute and relative frequencies; for metric variables median and range (minimum, maximum) are given as none of these variables was normally distributed. Differences between the professional groups were assessed by Mann-Whitney U or Chi-Squared test according to variable level. For several outcome measures, differences between the operative and anesthetic staff within the two professional groups were also tested. Spearman correlation was used to assess associations between measures of subjective and objective knowledge. Since the study was not hypothesis-driven, all analyses are of a purely exploratory nature. In all analyses, a p-value of less than 0.05 was considered statistically significant. All analyses were conducted using SPSS version 21.

## Results

### Checklist use

In 2011, one month after the first test run the SSC was used in 77.1% (135/175) of operations; within used SSCs, 36.3% (49/135) were complete and 63.7% (86/135) were partially complete. According to the second test run, the SSC was used in 99.2% (129/130) of operations, however only two (1.6%) of those were completed.

**Fig 2:**
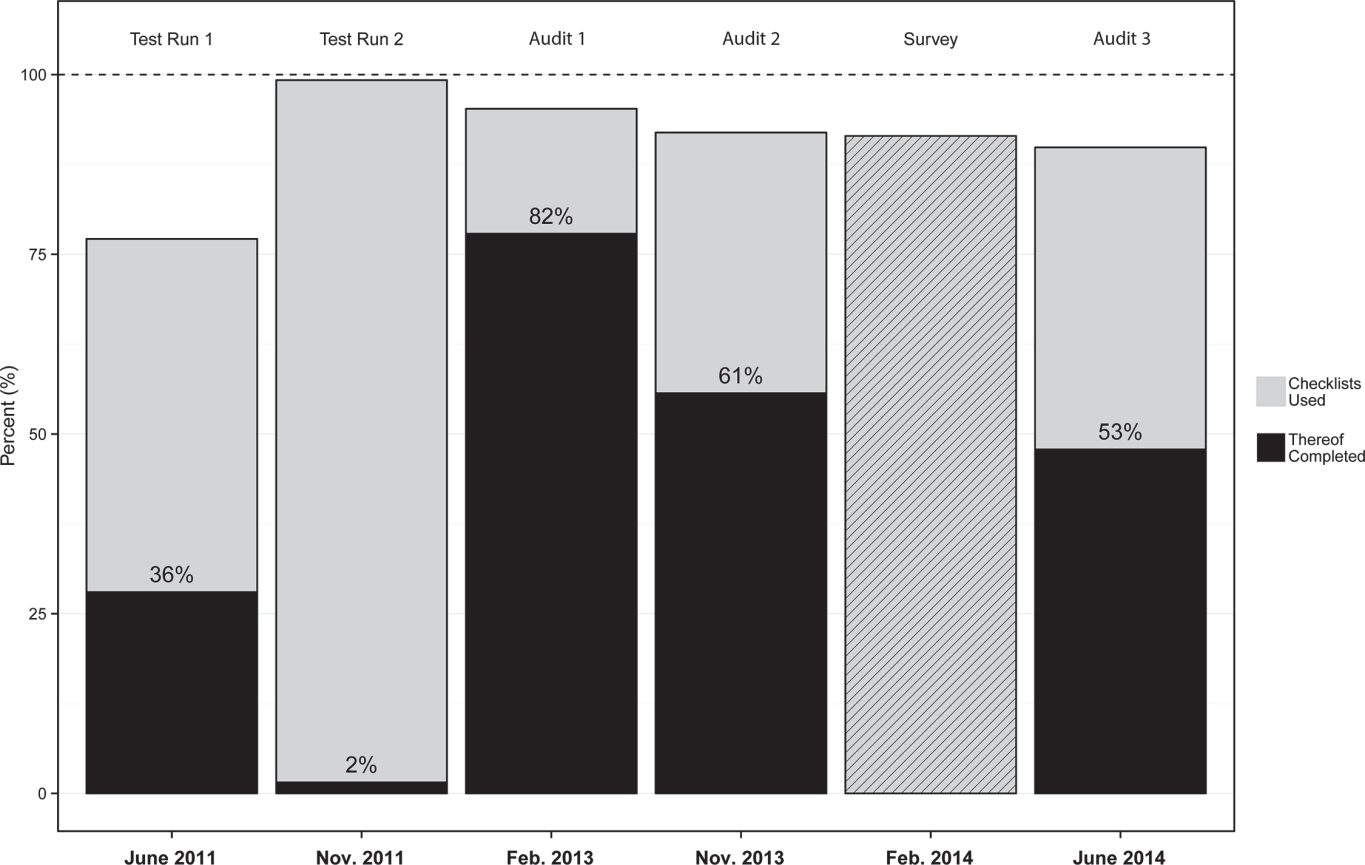
Utilization and completion rates of the SSC.

After introducing the SSC into all operating theaters, three unannounced audits took place. Audit 1 showed that the SSC was used in 95.3% (241/253) of operations; within used SSCs, 81.7% (197/241) were complete and 18.3% (44/241) were partially complete. Audit 2 showed that the SSC was used in 91.9% (251/273) of operations; within used SSCs, 60.6% (152/251) were complete and 39.4% (99/251) were partially complete. According to the third audit, SSCs were used in 89.9% (231/257) of operations; within used SSCs, 53.2% (123/231) were complete and 46.8% (108/231) were partially complete (Fig. 2).

In partially completed SSCs of test run 1, checkbox completion varied from 79% (68/86) to 97% (83/86). SSC items such as “hygiene”, “site marked”, “informed consent”, “introduction of team members” and “further questions” were least frequently checked. In test run 2, lower completion rates were observed for almost all SSC items (Table 2).

**Table 2:** Quantitative analyses of compliance in partially completed SSCs during test run 1 and 2.

Spectrum of checkboxes ticked within incomplete SSCs	Jun-2011	Nov-2011
	N (%)	N (%)
Sign In	n = 86	n = 127
- Patient Name?	74 (86)	113 (89)
- Date of birth?	74 (86)	113 (89)
- Measures regarding hygiene necessary?	68 (79)	46 (36)
- Site marked?	73 (85)	81 (64)
- Informed consent for surgical procedure applicable?	71 (83)	33 (26)
- Antibiotic prophylaxis?	72 (84)	81 (64)
- Informed consent for anesthetic procedure applicable?	72 (84)	91 (72)
- Known allergies?	72 (84)	91 (72)
Team Time Out		
- Introduction of team members?	81 (94)	83 (65)
- Patient Name?	80 (93)	116 (91)
- Date of birth?	80 (93)	116 (91)
- Type of procedure?	81 (94)	116 (91)
- Site of operation?	83 (97)	117 (92)
- Anticipated critical events?	80 (93)	107 (84)
- Imaging applicable?	80 (93)	116 (91)
- Further questions?	77 (90)	77 (61)
Sign out		
- Name of the procedure and deviations, if any?	78 (91)	94 (74)
- Sponge counts correct?	78 (91)	108 (85)
- Instrument counts correct?	78 (91)	108 (85)
- Specimen is labelled?	73 (85)	79 (62)
- Were there any problems with the equipment?	76 (88)	89 (70)
- Relevant information for the post-operative procedure?	77 (90)	103 (81)

### General survey results

In February 2014, 891 employees were asked to participate in an online survey and the overall response rate was 18.4% (164 returned questionnaires). 4 replies had to be excluded from analysis due to the respondents’ unknown affiliation with either professional group of interest. This left questionnaires from 60 consultants (37 surgeons and 23 anesthesiologists) and 100 nursing staff (32 nurse anesthetists and 68 operating theater nurses) to be evaluated (Table 3).

**Table 3:** Online survey – sample characteristics.

		Consultants	Nursing staff
N (%)		60 (37.5)	100 (62.5)
Female (%)		28 (46.7)	79 (79.0)
Median age in years (range)		46.5 (28, 59)	36 (21, 60)
Professional experience (%)	0 – < 5 years	6 (10)	21 (21.0)
	10 – < 20 years	38 (63.3	52 (52.0)
	More than 20 years	16 (26.7)	27 (27.0)
Hours spent in the OR in an average week	0 – < 16	12 (20.0)	21 (21.0)
	16 – < 32	11 (18.3)	25 (25.0)
	32 – and more than 40	37 (61.7)	54 (54.0)

The sample consisted of 66.9% (107/160) women and 33.1% (53/160) men. The median age was 40.5 with a range from 21 to 60. 58.8% (94/160) of responders had been working in an operating theater for at least 10 years and 56.9% (91/160) reported to spend at least 32 hours per week there.

### Frequency of checklist utilization

91.3% (146/160) confirmed to use the SSC in their operations and 80.6% (129/160) specified having used the SSC in 91 – 100% of all operations (Table 4). Overall, consultants’ estimation of how often they were using the SSC was significantly higher (p = 0.038) than that of the nursing staff. Further analysis in the respective subgroups revealed significant differences between surgeons and anesthesiologists (p = 0.006) but not between nurse anesthetists and operating theater nurses (p = 0.878) (S1 Fig.).

**Table 4:** Results of the online survey.

		Consultants	Nursing staff
		N = 60 (37.5%)	N = 100 (62.5%)
Use of SSC (%)	Yes	59 (98.3)	87 (87.0)
Type of SSC (%)	WHO-SSC	29 (49.2)	54 (62.1)
	Universal Protocol	3 (5.1)	0 (0)
	Swiss Protocol	16 (27.1)	15 (17.2)
	None of these	11 (18.6)	18 (20.7)
Frequency of SSC use	0–70% of operations	1 (1.7)	14 (14.0)
	71–90% of operations	6 (10.0)	10 (10.0)
	91–100% of operations	53 (88.3)	76 (76.0)
Satisfaction with SSC	Very satisfied and satisfied	45 (75.0)	54 (54.0)
	Somewhat satisfied	9 (15.0)	30 (30.0)
	Rather and very unsatisfied	6 (10.0)	16 (16.0)
Subjective knowledge	Very good and rather good	50 (83.3)	82 (82.0)
	Okay	9 (15.0)	10 (10.0)
	Rather bad and very bad	1 (1.7)	8 (8.0)
Median number of correctly answered questions (range)		8 (4, 10)	8 (3, 9)

### Satisfaction with checklist implementation

Overall satisfaction with the implementation of the SSC within all professional groups was high (Table 4). On the whole, consultants were more satisfied with the implementation process of the SSC than were nursing staff (p = 0.021). Again, significant differences were found between surgeons and anesthesiologists (p = 0.013) but not between nurse anesthetists and operating theater nurses (p = 0.108) (S2 Fig.).

### Knowledge concerning the SSC

Generally, subjective knowledge about the SSC and its usage was self-reported as high within all professional groups. 82.5% (132/160) announced that their knowledge is very good or rather good (Table 4). On average, consultants’ estimation of subjective knowledge was significantly higher (p = 0.016) than estimation amongst nursing staff. Differences between surgeons and anesthesiologists proved significant (p = 0.006), whereas no significant differences among the two subgroups of nursing staff could be determined (p = 0.174) (S3 Fig.).

With regard to objective knowledge, measured as the total number of correctly answered true/false questions, in median 8 out of 10 questions (minimum 3, maximum 10) were answered correctly. No significant differences between professional groups (p = 0.056) or among consultants (p = 0.669) and nursing staff (p = 0.644) could be determined.

Furthermore, no significant correlations between subjective and objective knowledge were found for either consultants (rho = -0.134, p = 0.307) or nursing staff (rho = -0.178, p = 0.077) as well as within the total cohort (rho = -0.131, p = 0.098).

## Discussion

Each of the surgical departments in our hospital implemented the SSC into their routine procedures. Results of audits and the online survey were found to be in accordance with respect to the rate of SSCs used and subjective belief of healthcare professionals. However, the rate of completed SSC continuously decreased over time. As summarized by Treadwell [23], barriers to SSC implementation generally consist of confusion regarding the proper use of the checklist, pragmatic challenges to efficient work flow, individual beliefs and attitudes. Our data have shown that the implementation of a new tool is a continuous process that requires the willingness of healthcare professionals to participate as well as the expertise to implement and constantly improve such a tool.

Between test run 1 and 2, the rate of completed SSCs decreased from 36.3% to 1.6% within months. This was unexpected since much effort was put into developing an SSC together with all affected team members. Furthermore, regular focus group discussions, trainings and on-site visits in the operating room were performed by the Department of Quality and Risk Management to best support all healthcare professionals working with the SSC.

During the SI, 8 items were infrequently completed such as ‘hygiene’, ‘site marked’, ‘informed consent’ and ‘antibiotic prophylaxis’. As these items are crucial to enhance patient and employee safety, noncompliance was incomprehensible and its reasons remained unclear at that point. During the TTO, checkpoint items such as ‘introduction of team members’ and ‘further questions’ were also infrequently completed. This led to the assumption that these items could have been inconvenient for the operating team. Non-structured interviews confirmed that the operating team did not consider these questions to be helpful during the TTO procedure. Furthermore, the team felt uncomfortable with answering them when among familiar staff.

The test run in the pilot unit showed the need to provide additional information to all healthcare professionals before the SSC could be rolled out to all surgical departments. As part of this information process, we explained that introducing the team members by name, can be of great benefit by reminding already familiar teams of the members’ names, by helping new members to feel accepted into the team and by generally flattening the hierarchy in the operating theater [19]. During the first test run the SSC was also foreseen to be signed by the team members who are responsible for their SSC elements in order to demonstrate ownership. However, nearly all signatures were missing. Therefore, it was defined to adjust the SSC for the second test run to only note the name of the operating theater, the date of the operating procedure and the patient’s adhesive label. We also reminded the checklist coordinator to thoroughly tick the checkpoints once a checkbox item was discussed. As it can be seen by our results, the checklist coordinator strictly followed this rule as we did not produce 100% checked SSC over time.

The stepwise implementation of the SSC to all surgical departments was optimized and consisted of three main points: focus group discussions with healthcare professionals, role play and introduction of contact persons in surgical departments. In focus group discussions, healthcare professionals of surgical departments were guided by a moderator from the Department for Quality and Risk Management. The aim and the goal of the adapted SSC as well as each item of the SSC was thoroughly discussed and questions were addressed directly in this or further meetings. All focus groups revealed the SSC as a useful tool as it helps to create a safe environment in the operating setting. However, the implementation of a checklist in general needs time to adjust common procedures and habits. Therefore, in role plays we demonstrated that checklist use is not trivial and needs to be trained. Such trainings were offered to all surgical departments; however, some declined these trainings. This can be interpreted in two ways, i) healthcare professionals feel to use the SSC as supposed and decline further trainings or ii), they do not accept external support as given by the Department for Quality and Risk Management.

All new measures taken together supported the further implementation process and resulted in a higher compliance rate (95.3% in Feb. 2013) which was more or less stable over the observational period (91.9% in Nov. 2013, 89.9% in June 2014). The encouragement of the checklist coordinator to tick the checkboxes thoroughly, initially resulted in a high proportion of completed SSCs, however, was followed by a decrease over time from 60.6% to 53.2%. The proportion of completed SSCs in audit 1 was self-reported by healthcare professionals and showed the necessity of independent control mechanisms. Starting with audit 2, objective evaluations were introduced and painted a different picture of SSC compliance. Nevertheless, comparing our results to international data, these figures for a short period of SSC use are comparable to or even higher than those reported by other hospitals [6, 11, 24–26].

We also introduced unannounced on-site visits; however, described as the Hawthorne effect, observational results were likely biased as teams tend to follow procedures more rigorously when they know they are being observed [27]. We therefore focused on unannounced audits and forwarding feedback to department leaders. However, giving real-time feedback of SSC compliance results did not influence the behavior of healthcare professionals. This led us to believe that individual attitudes of staff towards the checklist played a major role in the outcome of the SSC utilization [23].

Considering the fact that 47,000 operations are done per year within our hospital, according to the SSC compliance rate, approximately 10% of all operations are still performed without the use of an SSC. As it is our priority to reach 100% SSC compliance and since we were eager to receive further hints on process improvement, we gave all healthcare professionals working in an operating theater the opportunity to take part in an online survey assessing subjective and objective knowledge of as well as satisfaction with the SSC implementation.

The introduction of new tools like an SSC often evokes criticism, as habits have to be changed. Thereby it can be positively interpreted that more than 60% were satisfied with the SSC implementation. Consultants were more satisfied with the SSC implementation. This result was unexpected, as nursing staff have always been the most supportive members in a theater team [27]. However, from a subjective point of view, during the introduction period more effort was dedicated towards consultants, and amongst those, especially to surgeons to overcome general resistance to change their habits. Therefore, survey data support the need to focus on all healthcare professionals during an implementation phase.

Subjective knowledge was self-rated as being outstanding, however, considering that the SSC has been part of their routine procedure for more than three years, objective knowledge was not impressive and needs further improvement. To overcome this lack of knowledge, training has been found to best raise the compliance to the SSC [24] and was also mentioned as an important improvement tool in the comment section (S1 Table) of the online survey.

In general, we also recognized that our expertise to perform SSC-trainings or focus group discussions increased over time. Therefore, we have to admit that we were not well prepared for the initial phase of the SSC implementation. From a retrospective point of view we strongly recommend all SSC-beginners not to only screen the literature and adapt the SSC as proposed by the WHO, but rather to visit a hospital, where the SSC is successfully implemented. Also, sharing as many SSC-opportunities in an operating room as possible helps to learn how to best train others in using the SSC. In advance, discussions with healthcare professionals who support and refuse the SSC is also a helpful method in order to prepare oneself as a trainer or project leader who will be responsible to implement the new tool. We also recommend setting up a distinctive SSC program prior implementation which should contain following items: i) set up a learning program, ii) develop a system for continuous evaluation, and iii) implement a feedback system.

Results from the audits as well as the online survey showed that further improvements are needed to achieve better compliance and through it acceptance of the SSC. In terms of lessons learned over the last years, we have to report a bundle of improvements. First, attitudes of staff towards the SSC influenced the willingness to participate in trainings. Therefore, hands-on-leadership is needed to overcome this hurdle as it is the most powerful predictor of successful SSC implementation and acceptance [29]. Second, to assure proper use of the SSC, training should become an integral part within each surgical department and has to be performed continuously. To further facilitate the use of the safety tool, we aim to produce a video with proponents. This video is to become an integral part of trainings for operating theater teams and medical students. Third, local contact persons within each surgical department should be empowered to organize trainings or initiate audits when needed to reinforce the use of the SSC within their environment [28]. They should also receive additional trainings by experts to best enable them to support their colleagues in the operating theater. Fourthly, training needs to be performed by experts; in our case the expertise evolved over the period of implementing the SSC. Finally, to demonstrate good practice, study data and case studies where the SSC enhanced patient safety should be published within each hospital, rather than solely communicating SSC non-compliance.

A limitation of the current study was the poor response rate of the online survey. One reason might have been that this survey was the first being performed online within our hospital. Employees reported certain concerns regarding anonymity, though the survey process was outlined in detail. Another reason for the poor response rate could have been the peculiar fact that 25–50% of all employees within our hospital had not yet activated their email account, which was found out after the survey was performed. Although the response rate is comparable to Mascherek et al. [20], we could have probably augmented it by sending out at least two or three reminders to non-responders [30]. Another limitation was the fact that there was no baseline survey prior to the implementation of the SSC. Additionally, we assessed compliance in terms of SSC use; however, we did not investigate the quality of SSC use. Finally, the study lacks information on whether training was done inadequately and therefore might have had any influence on outcome measures.

## Conclusion

In conclusion, we found that the combined approach of assessing compliance and subjective and objective knowledge appeared to be a useful instrument to investigate the implementation of safety tools such as the SSC. The main key in increasing SSC use is a combined strategy of repetitive training and assessment on the part of the involved healthcare professionals [29].

## Supporting Information

S1 Fig Frequency of checklist use per specialty group of consultants and nursing staff, numbers above bars represent absolute frequencies. (TIF)

S2 Fig Satisfaction with checklist implementation per specialty group of consultants and nursing staff, numbers above bars represent absolute frequencies. (TIF)

S3 Fig Subjective knowledge about checklist use per specialty group of consultants and nursing staff, numbers above bars represent absolute frequencies. (TIF)

S1 Table 64 annotations were given in the online survey, thereby, improvements were suggested and multiple answers had been - Reduce the number of questions on the SSC
- Install an electronic version of the SSC
- Implement the SSC also in outpatient departments
- Offer additional SSC-training
(DOCX)
