# Correction: Implementation of a Surgical Safety Checklist: Interventions to Optimize the Process and Hints to Increase Compliance

**DOI:** 10.1371/journal.pone.0123726

**Published:** 2015-04-01

**Authors:** The PLOS ONE Staff

The third author is listed incorrectly in the citation for this article. The correct citation is given below.

Sendlhofer G, Mosbacher N, Leitgeb K, Kober B, Jantscher L, Berghold A, et al. (2015) Implementation of a Surgical Safety Checklist: Interventions to Optimize the Process and Hints to Increase Compliance. PLoS ONE 10(2): e0116926. doi:10.1371/journal.pone.0116926
