# Supplementary figures and images for: Implementation of a Surgical Safety Checklist: Interventions to Optimize the Process and Hints to Increase Compliance
Source: PLoS One. 2015 Feb 6;10(2):e0116926. doi: 10.1371/journal.pone.0116926 (PMC4319744; doi:10.1371/journal.pone.0116926)

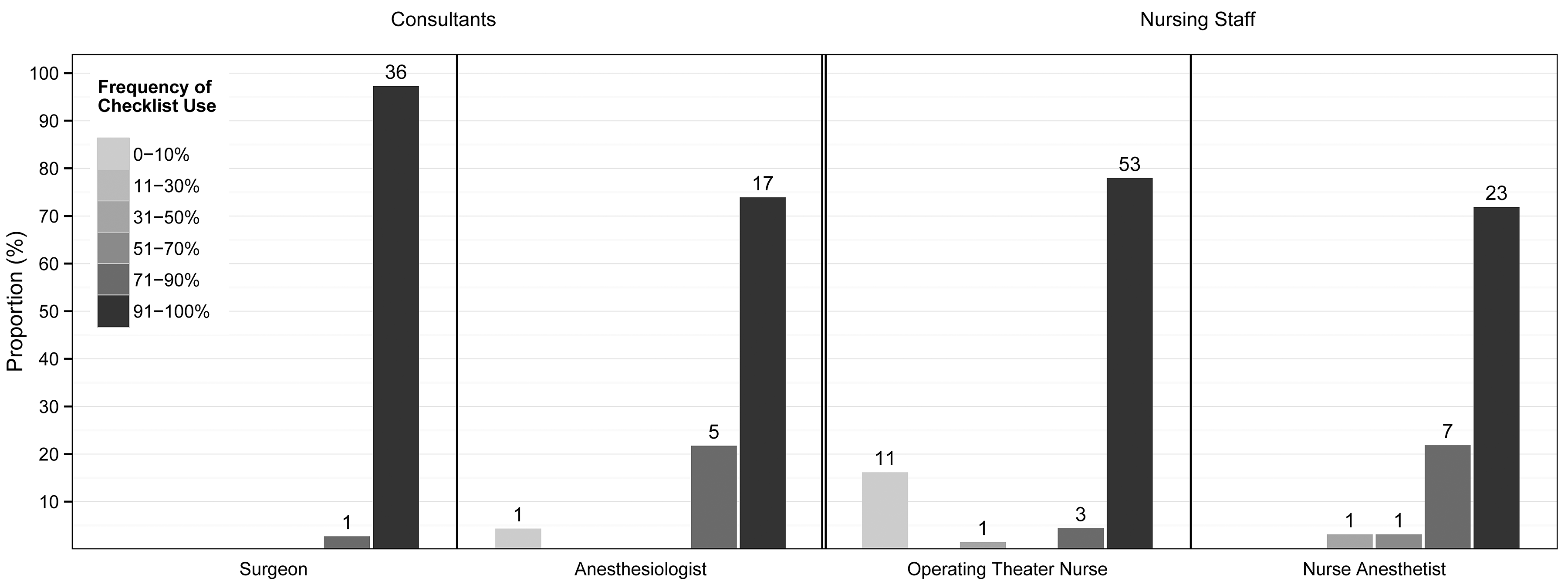

Supplement: S1 Fig — (TIF) [file pone.0116926.s001.tif]

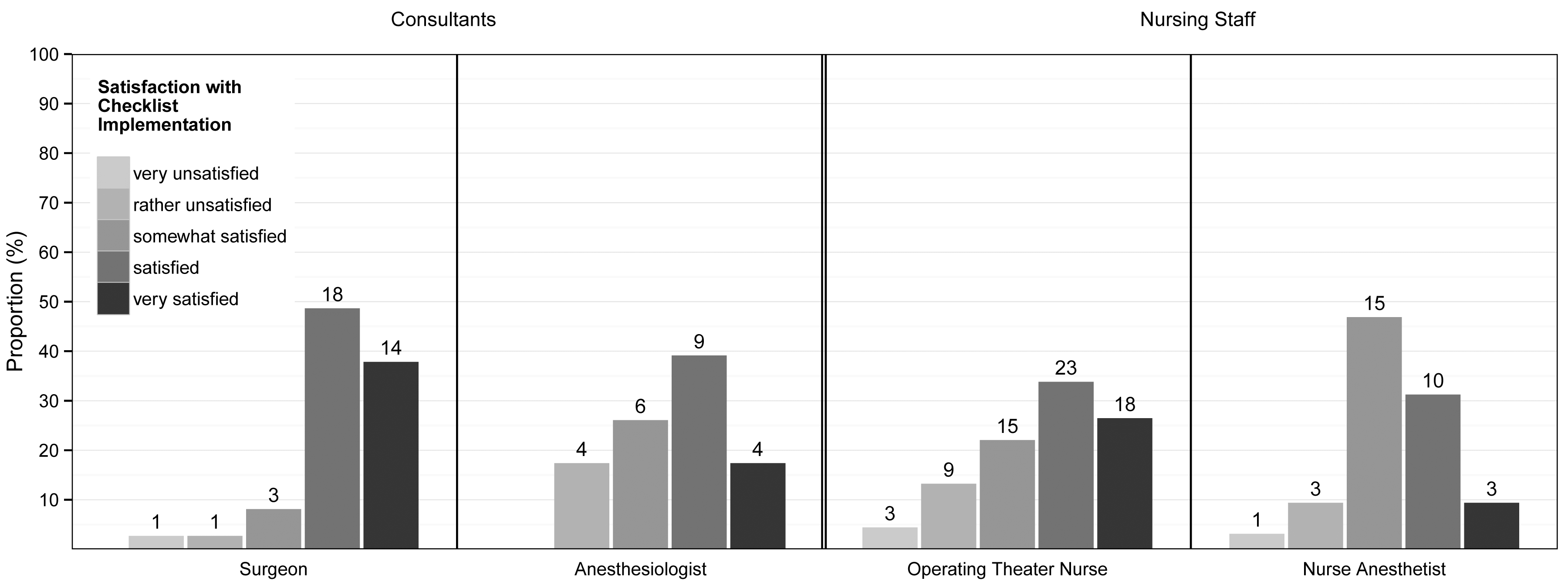

Supplement: S2 Fig — (TIF) [file pone.0116926.s002.tif]

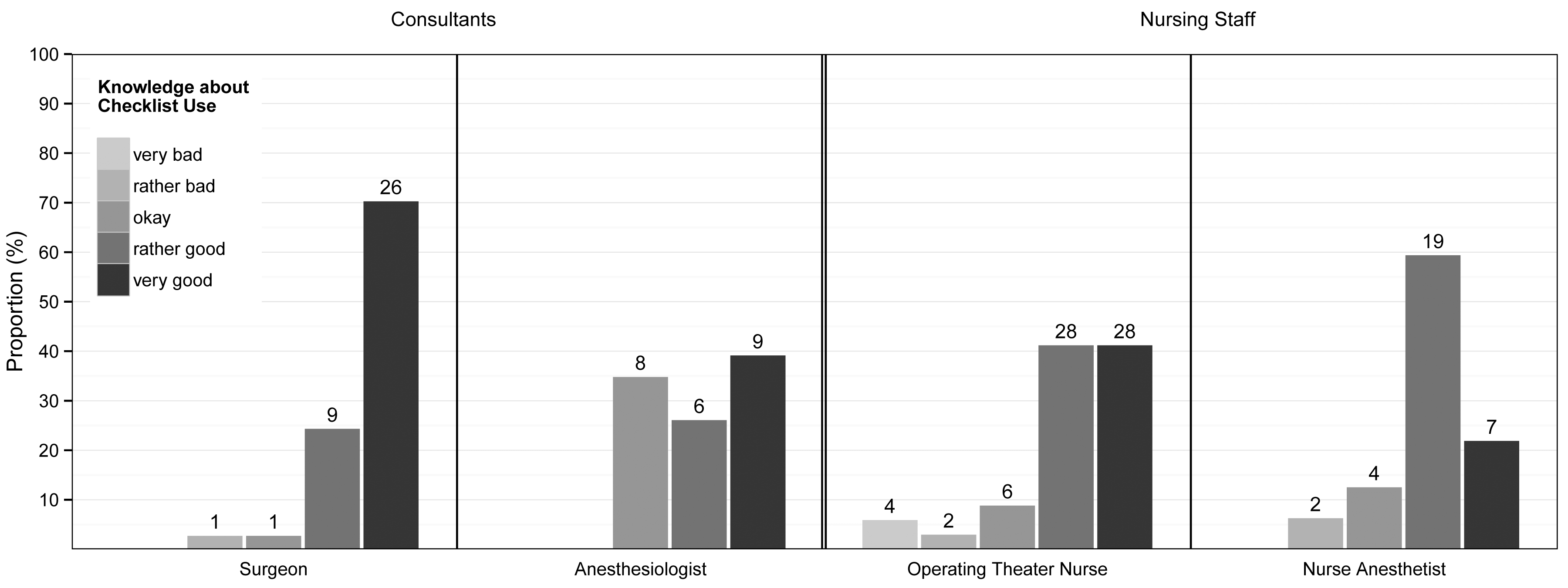

Supplement: S3 Fig — (TIF) [file pone.0116926.s003.tif]
