# Supplementary material for: Implementation of a Surgical Safety Checklist: Interventions to Optimize the Process and Hints to Increase Compliance
Source: PLoS One. 2015 Feb 6;10(2):e0116926. doi: 10.1371/journal.pone.0116926 (PMC4319744; doi:10.1371/journal.pone.0116926)
Supplement: S1 Table — - Reduce the number of questions on the SSC - Install an electronic version of the SSC - Implement the SSC also in outpatient departments - Offer additional SSC-training (DOCX) [file pone.0116926.s004.docx]

***Supplementary notes***

The survey allowed for annotations to be added. 64 improvements were suggested and multiple answers included:

- reducing the number of questions on the SSC
- making an electronic version of the SSC
- implementing the SSC into outpatient departments
- offering additional training on the SSC
